# A case of chronic spontaneous urticaria with wheals lasting for more than a week

**DOI:** 10.1111/1346-8138.17480

**Published:** 2024-09-30

**Authors:** Satoshi Morioke, Masaya Moriwaki, Akio Tanaka, Michihiro Hide

**Affiliations:** ^1^ Department of Dermatology, Graduate School of Biomedical and Health Sciences Hiroshima University Hiroshima Japan; ^2^ Department of Dermatology Hiroshima City Hiroshima Citizens Hospital Hiroshima Japan

**Keywords:** drug eruptions, erythema, exanthema, pruritus, vasculitis

## Abstract

Urticaria is characterized by the development of wheals which usually disappear within a day, and are not present beyond a few days. A 58‐year‐old man began to develop edematous, partially ring‐shaped erythematous lesions accompanied by severe body itching without any particular cause, approximately 2 months before his first visit to our hospital. Each rash emerged as being about 5 mm in diameter, gradually enlarging over several days, and disappeared in up to 10 days. Despite oral treatment with several antihistamines and 10 mg of prednisolone, there was no improvement. Most eruptions disappeared without a trace, but the erythema that appeared on the palms left desquamation. The patient had a history of shellfish allergy, but otherwise no atopic diseases. Drug eruption was ruled out due to a lack of regular taking other medications. Histopathological findings of the skin lesions showed moderate lymphocytic and few eosinophilic infiltrates with edema, but no evidence of vasculitis. Despite the concomitant use of two second‐generation antihistamines and montelukast, the rash did not improve. The symptoms began to improve following oral intake of 1.5 mg of betamethasone, which was tapered off with the addition of 150 mg of cyclosporin. The use of all medications was stopped at 4 months from the first visit without recurrence. Wheals of chronic spontaneous urticaria may last for longer than a week without apparent histopathological findings of vasculitis.

## 1 INTRODUCTION

Chronic spontaneous urticaria (CSU) causes localized and temporary edema in the dermis, resulting in wheals of various sizes and shapes, and causing itching and possibly burning sensations. However, it usually disappears without leaving any traces within 30 min to 24 h.^1^ Urticarial vasculitis (UV) is an important differential diagnosis with edematous erythema and postinflammatory hyperpigmentation that persist for >24 h. A survey of 883 physicians from 92 countries revealed that the key features in confirming the diagnosis of UV include wheals lasting >24 h (72%), and skin biopsy results (63%), postinflammatory pigmentation (46%). Complete blood count and serum C‐reactive protein tests might be useful.^2^

We experienced a case of wheals with markedly prolonged infestation that gradually expanded over several days and disappeared in up to 10 days. Nevertheless, no obvious inflammatory reaction and no histological signs of vasculitis were observed.

## 2 CASE REPORT

**FIGURE 1:**
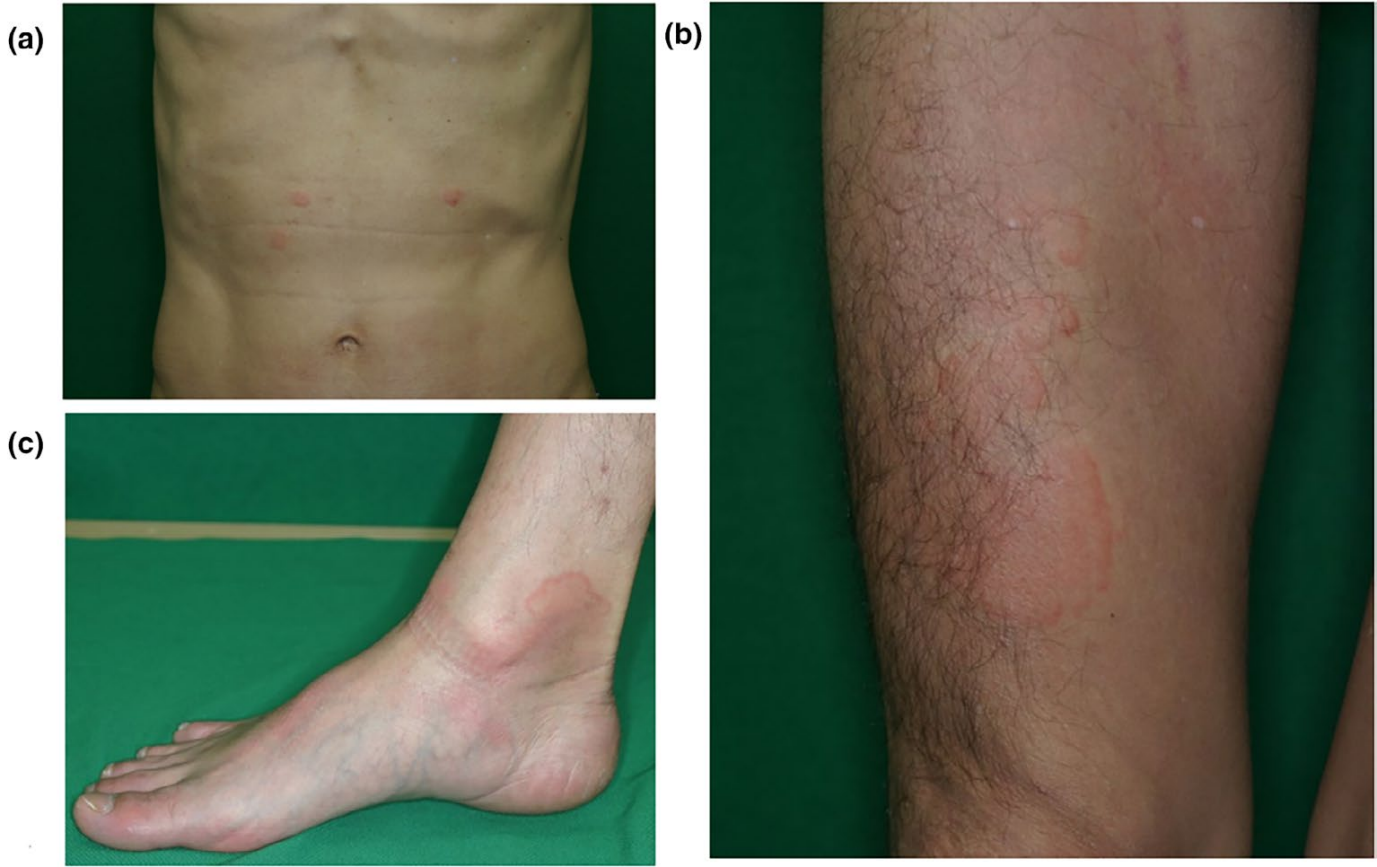
Clinical presentation on the first visit to our department. (a) Red bean‐sized edematous erythematous spots scattered on the trunk. (b) Scattered edematous erythematous patches on the right thigh ranging from the size of the thumb to the size of a walnut. (c) Part of the right foot with annular erythema. Each rash gradually expanded to the size of the palm over several days and disappeared after 7–10 days.

**FIGURE 2:**
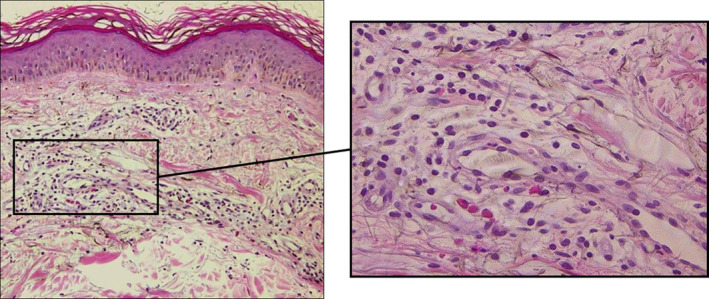
A biopsy was performed from an edematous erythema on the right lower leg. No significant changes in the epidermis were observed. Moderate lymphocytic infiltration and edema in the perivascular and interstitial areas of the superficial dermis were noted, and a few eosinophils were found, but no nuclear dust or fibrinoid degeneration of the vessel wall was found.

**FIGURE 3:**
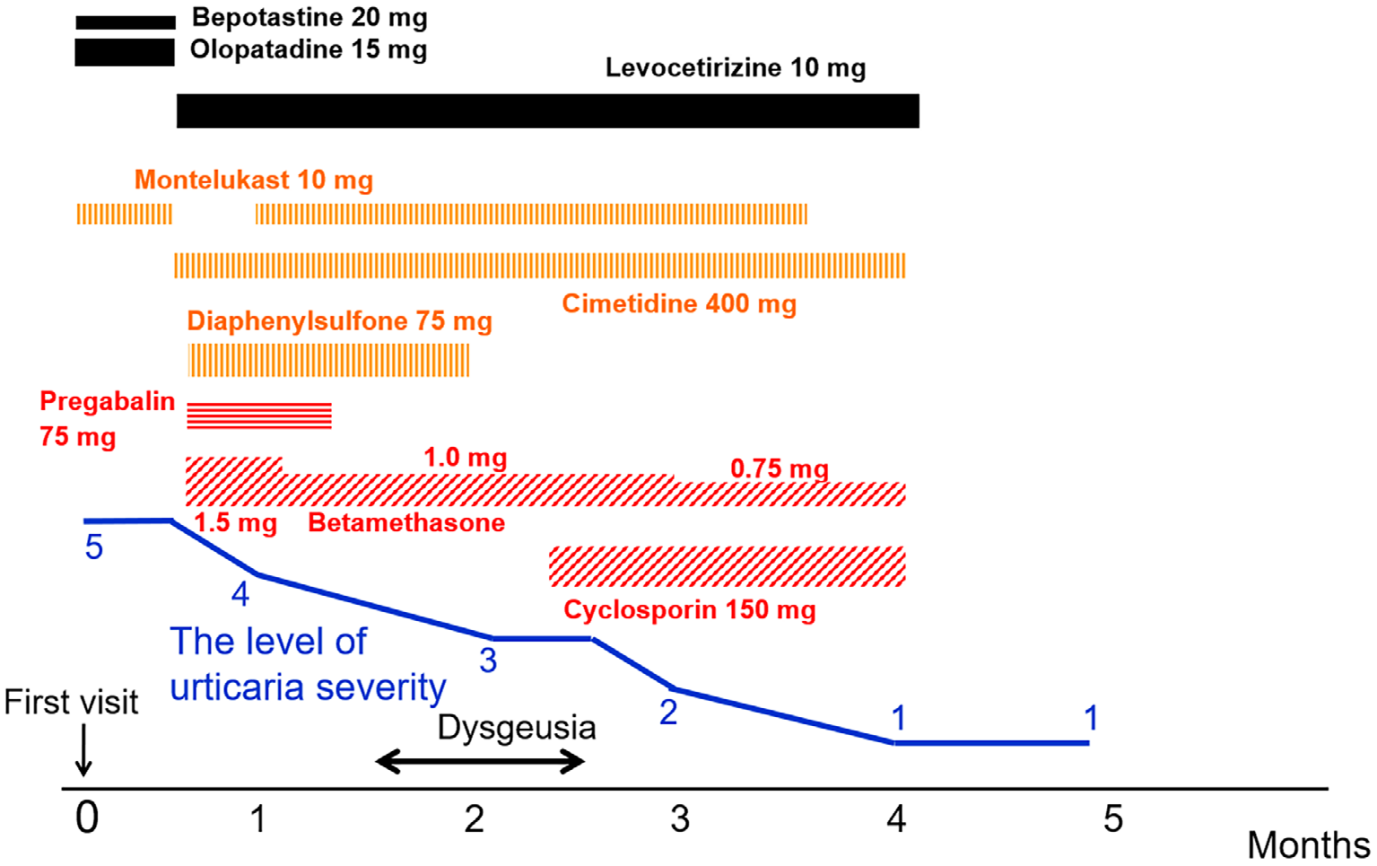
Clinical course after the start of treatment. The severity of urticaria was expressed using the severity level listed in the Japanese guideline for urticaria management.^3^ These severity levels are as follows: 5, unable to have a social life; 4, barely able to live with disabilities; 3, the discomfort is tolerable; 2, symptoms that are not noticeable; 1, no symptoms. With oral betamethasone and cyclosporine, the number of wheals reduced and the pruritus disappeared. Five months after the initial visit, the patient's urticaria was declared to be cured.

A 58‐year‐old man with a 2‐month history of edematous, partially ring‐shaped erythematous lesions accompanied by severe itching, was referred to our department. He had rashes throughout his body, without any apparent cause. Each rash emerged as about the size of a red bean, which gradually enlarged over several days, and disappeared in up to 10 days without leaving any scaling or pigmentation, except for the palms which showed mild desquamation. He received several antihistamine medications and 10 mg of oral prednisolone, but the rash did not improve. He had no particular family history. He had a history of allergy to shellfish, but otherwise no other atopic background. On initial examination, bean‐sized edematous erythematous patches (Figure 1a) were observed on the trunk. Edematous erythema grew up to the size of the palm and some with annular erythema were also scattered on his extremities (Figure 1b,c). Laboratory data showed white blood cells 4800/μL, red blood cells 435 × 10^4^/μL, hemoglobin 14.8 g/dL, platelets 19.5 × 10^4^/μL, C‐reactive protein 0.02 mg/dL, D‐dimer 1.3 μg/mL, immunoglobulin G (IgG) 833 mg/dL, immunoglobulin A (IgA) 150 mg/dL, immunoglobulin M (IgM) 72 mg/dL, complement component 3 65 mg/dL, component 4 23 mg/dL, and antinuclear antibodies <×80. No kidney or liver functional abnormalities were observed. A biopsy specimen from the erythema revealed no significant changes in the epidermis. Moderate lymphocytic infiltration and edema were noted in perivascular and interstitial layers of the superficial dermis, and a few small eosinophils were also observed. However, no findings suggestive of vasculitis, such as nuclear dust or fibrinoid degeneration of the vessel wall, were observed (Figure 2). Based on the above‐mentioned information, we made a diagnosis of atypical CSU and treated according to the algorithm suggested by the Japanese guideline for the management of urticaria.^3^ His clinical course is shown in Figure 3. Wheals accompanied by severe itching seriously interfered with his social life, making it difficult for him to concentrate on work. After his first visit, high doses of bepotastine (two‐fold) and olopatadine (three‐fold) in combination with 10 mg of montelukast were started orally, but no improvement was observed. The antihistamines were changed to a 2‐fold dose of levocetirizine (10 mg), and 400 mg of cimetidine and 75 mg of diaphenyl sulfone were used as supportive drugs. In addition, 1.5 mg of betamethasone was used in combination with pregabalin as a trial treatment for severe pruritus. Consequently, the itching was slightly reduced, but remained constant, and we thus restarted montelukast. After 1 month, the dose of betamethasone was reduced to 1.0 mg. After 1.5 months, he experienced dysgeusia without any particular cause and became able to taste only bitterness. Considering the adverse effects of the drugs, pregabalin and diaphenyl sulfone were discontinued, but the dysgeusia did not improve. Meanwhile, the individual lesions gradually became smaller and less itchy. The duration of them was somewhat shorter than initially observed, but still long, about a week, but dysgeusia improved as the disease progressed. Following 10 weeks use of betamethasone, 150 mg of cyclosporine was added, and the betamethasone dose was then reduced to 0.75 mg. The number of newly development of wheals and their size, duration, and itch all continuously decreased and eventually disappeared. After 5 months from the initial diagnosis, the patient was judged to be cured.

## 3 DISCUSSION

In many cases of urticaria, wheals disappear within 24 h, but wheals persist for >24 h in 20.8% of patients and in 8.8% for >48 h.^4^ The international guideline^1^ for the management of chronic urticaria suggests that even if the average duration of the wheal exceeds 24 h, if no evidence of vasculitis is found on skin biopsy, it should be treated as urticaria. However, to our knowledge, no studies have reported CSU characterized by wheals lasting longer than a week. In this case, wheals initially persisted for up to 10 days, which is far different from the common characteristics of urticaria. In Japan, this type of rash has traditionally been called “urticarial erythema”, but this is merely a provisional name and cannot be said to be internationally recognized. Differential diagnosis for such cases with long‐lasting wheals includes UV, erythema multiforme, erythema gyratum repens, and Schnitzler syndrome. In the present case, the patient had no fever, bone pain, arthralgia, or increase of serum IgM, therefore ruling out Schnitzler syndrome. The shape of skin eruptions was not multilayered grain‐like or serpentine, and thus erythema gyratum repens was unlikely. Histopathological findings showed no evidence of vasculitis, liquefaction degeneration at the epidermal‐dermal interface, or keratinocyte necrosis, and thus erythema multiforme was ruled out. Nevertheless, moderate lymphocytic infiltration and edema were observed around blood vessels and in the interstitium of the shallow dermis. A subtype of urticaria, i.e., delayed pressure urticaria, is characterized by wheals that last for longer than 24 h and histopathologically conspicuous inflammatory cell infiltration in the dermis.^5^, ^6^ In the present case, the patient had no episode of wheal formation in response to pressure, but moderate inflammatory cell infiltration in the dermis may have contributed to the prolonged duration of wheals. CSU and UV may intersect in cases with long duration of wheals, postinflammatory pigmentation, and systemic symptoms, etc.^7^ Guitart pointed out that even in cases characterized by a long‐lasting eruption, if no histological vasculitis was noted, it should be considered distinct from UV.^8^ This case had no obvious underlying disease and the rashes resolved over 7–10 days, but no histological evidence of vasculitis was observed, so there was no inconsistency in considering it urticaria. It is believed that some mechanism other than activation of mast cells and the associated release of vasoactive substances such as histamine is also involved in the formation of wheals in urticaria. In our case, it was estimated that the unknown mechanism had a long duration of action. Oral steroids and cyclosporine were taken to suppress the patient's symptoms, and eventually resulted in cure with 5 months of treatment. This case revealed that wheals of urticaria may last beyond a week, approximately up to 10 days without evidence of UV, and could be well managed according to the algorithm for CSU. Thus, urticaria may form a broader disease spectrum than previously thought. The eligibility of using medications such as corticosteroids and cyclosporin to suppress inflammation in the long‐lasting wheals should be further studied in more cases of urticaria with long‐lasting wheals. This case is rare and does not fit the general definition of urticaria, which is that each wheal disappears within a day, therefore, we believe it was necessary to report this case, collect similar cases around the world, and carry out further analysis.

## CONFLICT OF INTEREST STATEMENT

Authors declare no conflict of interests for this article.

## Data Availability

Data sharing is not applicable to this article as no datasets were generated or analyzed during the current study.
